# Imaging oxidative stress in brains of chronic methamphetamine users: A combined 1H-magnetic resonance spectroscopy and peripheral blood biomarker study

**DOI:** 10.3389/fpsyt.2022.1070456

**Published:** 2023-01-10

**Authors:** Sarah E. Watling, Samantha Jagasar, Tina McCluskey, Jerry Warsh, Shawn G. Rhind, Peter Truong, Sofia Chavez, Sylvain Houle, Junchao Tong, Stephen J. Kish, Isabelle Boileau

**Affiliations:** ^1^Institute of Medical Sciences, University of Toronto, Toronto, ON, Canada; ^2^Brain Health Imaging Centre, Centre for Addiction and Mental Health, Toronto, ON, Canada; ^3^Campbell Mental Health Research Institute, Centre for Addiction and Mental Health, Toronto, ON, Canada; ^4^Department of Psychiatry, University of Toronto, Toronto, ON, Canada; ^5^Department of Pharmacology and Toxicology, University of Toronto, Toronto, ON, Canada; ^6^Faculty of Kinesiology and Physical Education, University of Toronto, Toronto, ON, Canada; ^7^Defence Research and Development Canada, Toronto Research Centre, Toronto, ON, Canada

**Keywords:** methamphetamine, glutathione, oxidative stress, inflammation, magnetic resonance spectroscopy, substance use disorder

## Abstract

**Introduction:**

Preclinical data suggest methamphetamine (MA), a widely used stimulant drug, can harm the brain by causing oxidative stress and inflammation, but only limited information is available in humans. We tested the hypothesis that levels of glutathione (GSH), a major antioxidant, would be lower in the brains of chronic human MA preferring polysubstance users. We also explored if concentrations of peripheral immunoinflammatory blood biomarkers were related with brain GSH concentrations.

**Methods:**

20 healthy controls (HC) (33 years; 11 M) and 14 MA users (40 years; 9 M) completed a magnetic resonance spectroscopy (MRS) scan, with GSH spectra obtained by the interleaved J-difference editing MEGA-PRESS method in anterior cingulate cortex (ACC) and left dorsolateral prefrontal cortex (DLPFC). Peripheral blood samples were drawn for measurements of immunoinflammatory biomarkers. Independent samples *t*-tests evaluated MA vs. HC differences in GSH.

**Results:**

GSH levels did not differ between HC and MA users (ACC *p* = 0.30; DLPFC *p* = 0.85). A total of 17 of 25 immunoinflammatory biomarkers were significantly elevated in MA users and matrix metalloproteinase (MMP)-2 (*r* = 0.577, *p* = 0.039), myeloperoxidase (MPO) (*r* = –0.556, *p* = 0.049), and MMP-9 (*r* = 0.660, *p* = 0.038) were correlated with brain levels of GSH.

**Conclusion:**

Normal brain GSH in living brain of chronic MA users is consistent with our previous postmortem brain finding and suggests that any oxidative stress caused by MA, at the doses used by our participants, might not be sufficient to cause either a compensatory increase in, or substantial overutilization of, this antioxidant. Additionally, more research is required to understand how oxidative stress and inflammatory processes are related and potentially dysregulated in MA use.

## 1. Introduction

Methamphetamine (MA), a central nervous system (CNS) stimulant, is the third most used recreational drug world-wide (1). Approximately 6% of Canadians report life time use of MA (2) and use is increasing across Canada (3). In a longitudinal study, having a hospital diagnosis of a MA-related condition was associated with an approximately fivefold risk of all-cause mortality compared to that in the general population (4). Rising recreational MA use, use of amphetamine containing medications prescribed to children and adults with attention deficit hyperactivity disorder (ADHD), coupled with uncertainty over whether MA damages the human brain, underpin the need to better understand the biological consequences of MA in the human brain.

The notion that MA might damage the human brain is derived primarily from preclinical animal data showing that high doses of MA, or its related stimulant amphetamine, cause loss of brain dopamine neuronal markers (5), swollen axons and silver staining (considered to reflect neurodegeneration) (6) and increased astrogliosis and microgliosis (7, 8) that commonly accompany neurological insults. However, in human MA users, brain levels of only some dopamine neuronal markers are decreased (9) and evidence of gliosis (10) and actual loss of brain gray volume (11) has not yet been established. Growing evidence suggests that drug use elicits immune system dysfunction and many drugs may act indirectly on immune cells by altering the activity of the neuroendocrine axis and neurotransmitter signaling, but may also directly act on immune cell receptors and regulate their activities (12). Correspondingly, dysregulation of systemic immunoinflammatory responses with central and peripheral release of cytokines and other acute phase mediators have been implicated in the neurotoxic effects of chronic MA use [see (13) for review].

Although the precise mechanism(s) by which high doses of MA cause brain damage in preclinical studies remain uncertain, excessive oxidative stress is a likely candidate. Thus, in experimental studies some antioxidants (14–16) and overexpression of the enzyme superoxide dismutase (17) attenuated changes in brain caused by MA. Preclinical observations also suggest generally reduced brain levels of the major antioxidant glutathione (GSH), albeit data are not entirely consistent [decreased (16, 18–22); no change (23); increased (24, 25)].

To our knowledge, studies of oxidative stress in the brain of human MA users are limited. Our post-mortem brain study found elevated levels of two oxidatively damaged lipids (4-hydroxynonenal; malondialdehyde) in dopamine-rich striatum and also in (dopamine-poor) frontal cortex (26), and a trend for lower GSH in a MA subgroup that experienced severe dopamine loss (27). To our knowledge, there is only one magnetic resonance spectroscopy (MRS) report to date of markedly (by 35%) increased levels of GSH in a single brain region of MA-dependent subjects (28). However, the low concentrations at which GSH is present in the brain and the overlapping resonances from other metabolites make it challenging to measure GSH *in vivo*. Additionally, research (29) suggests the point resolved spectroscopy (PRESS) acquisition method [employed by Su et al. (28)] cannot reliably quantify GSH at physiological concentrations (≥4 mM). MEGA (MEscher–GArwood)-PRESS is capable of quantifying GSH < 4 mM in the brain and spectral editing techniques can be applied to MEGA- PRESS to remove the signal from other metabolites. Briefly, the J-difference editing technique (used in the present study and described below) uses the difference between two acquisitions (one with and without editing pulses) to measure the target signal (30). Furthermore, there are no studies to date up until now (to our knowledge) investigating the relationship between systemic immunoinflammatory markers and central markers of oxidative stress in methamphetamine use. Given the suggestive preclinical, but limited human data on GSH and MA, we employed J-difference-edited ^1^H MRS pulse sequence MEGA-PRESS (an acquisition method capable of quantifying GSH with higher accuracy) to establish whether chronic recreational MA use might be associated with low brain levels of GSH, as an indirect marker of oxidative stress. GSH was assessed in the anterior cingulate cortex (ACC) and dorsolateral prefrontal cortex (DLPFC) due to these regions’ suggested involvement in MA use (31) and to replicate previous research (28). As a secondary, exploratory analysis, we investigated the relationship between peripheral immunoinflammatory biomarker profiles and brain levels of GSH.

## 2. Materials and methods

### 2.1. Participants

After receiving approval for this study from the Centre for Addiction and Mental Health (CAMH) Research Ethics Board, research participants were recruited from the Greater Toronto Area using advertisements *via* the online advertisements, brochures, and newspapers. After providing written informed consent, research participants completed a screening assessment at CAMH. At screening, research staff obtained medical and alcohol/drug use history, completed a psychiatric interview [using the structured clinical interview for DSM-5 Axis I Disorders: SCID-I/NP (32)] and collected urine and hair samples to confirm MA and other drug use. Participants were included in the study if they were ≥19 years of age and MA users were required to meet DSM-5 criteria for MA-related substance use disorder (SUD). HC were only included if they tested negative for drugs of abuse at the initial screening visit. All participants were excluded if they had any significant medical conditions including neurological conditions or serious head trauma, Axis I psychiatric disorders (except for MA-related SUD and comorbid mood and anxiety disorder in the MA group), or MRI contra-indications. Medications, recreational drugs, and tobacco use were not exclusionary in the MA group (as long as MA was the primary substance in use). Participants were asked to avoid all substances including methamphetamine, nicotine, alcohol, and recreational drugs overnight. An overnight abstinence period from methamphetamine was chosen to investigate the effects of current methamphetamine use on the brain; we suspected the maximum methamphetamine response on the outcome measures would be at a time close to the last use of the drug, rather than at a time, for example, in extended abstinence [supported by previous research from our group (18)].

### 2.2. MRI session

On MRI scan day, urine toxicology (BTNX Inc. Pickering, ON, Canada), breath alcohol and expired carbon monoxide measurements were taken and questionnaires assessing craving and withdrawal were also administered to MA users. A description of questionnaires and cognitive tasks is available in Supplementary Table 1.

Magnetic resonance imaging scans took place in a Discovery MR750 3T scanner in the Brain Health Imaging Centre at CAMH for approximately 1.5 h. To minimize head movement, each participant was positioned at the center of the eight-channel head coil with soft padding around the head. Magnet homogeneity was adjusted using the manufacture automated shimming routine.

### 2.3. Blood samples

Participants provided peripheral blood samples on the day of their PET scan [part of a larger study (10)]. Venous blood was drawn into a 10-mL K_2_EDTA tube and left at room temperature for approximately 45 min before a 20 min centrifugation at room temperature. Plasma supernatant was then aliquoted and frozen at −80°C until analysis.

Circulating blood biomarkers were analyzed in duplicate using multiplexed 96-well MULTI-ARRAY^®^ and MULTI-SPOT^®^ V-plex Ultra-Sensitive Human Immunoassay plates from MesoScale Diagnostics, LLC (MSD^®^, Gaithersburg, MD, USA); electrochemiluminescence detection of each MSD SULFO-TAG was captured and quantified using SECTOR Imager*™* instrumentation (MSD, Gaithersburg, MD, USA), according to manufacturers’ protocols as reported previously (33). Specifically, this study measured 25 immunoinflammatory proteins–including cytokines/chemokines/receptors [inter-leukin (IL)-6, -8, -10, tumor necrosis factor (TNF)-α/β, interferon (IFN)-γ], Eotaxin, IFN-γ-induced protein (IP)-10, monocyte chemoattractant protein (MCP)-1,-4, macrophage-derived chemokine (MDC), macrophage inflammatory proteins (MIP)-1 α/β, thymus activation regulated chemokine (TARC)]; acute phase proteins [c-reactive protein (CRP); oxidative/lytic enzymes [matrix metalloproteinases (MMP)-1,2,3,9,10, myeloperoxidase (MPO)]; neurotrophins [brain-derived neurotrophic factor (BDNF), vascular endothelial growth factor (VEGF)].

The raw data were analyzed using the Discovery Workbench 4.0 software (MSD) by fitting signal from the control calibration curves to a four-parameter logistic model and then back-fitting the electrochemiluminescence signal from each sample to calculate the unknown concentration. The standard curves for all cytokines tested exhibited low variability and a dynamic range of greater than 3 logs. Calculated mean concentrations with a percentage coefficient of variance >25% were excluded from the analysis. Results for each marker in the multiplex were expressed in pg/mL against known standards.

### 2.4. MRS data acquisition and analysis

**FIGURE 1:**
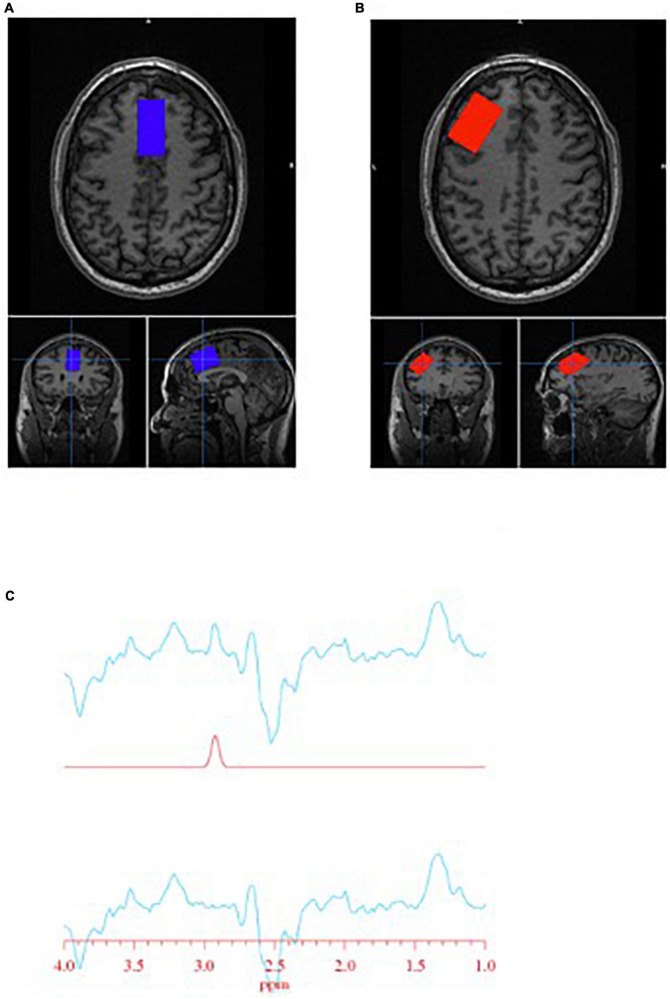
Glutathione (GSH) metabolite acquisition. **(A)** Voxel placement in the anterior cingulate cortex (ACC); **(B)** voxel placement in the left dorsolateral prefrontal cortex (DLPFC); and **(C)** GSH spectra obtained at 2.95 ppm.

Spectra were obtained on a 3T GE Discovery MR750 (SW: DV26 R01) scanner from two regions of interest: anterior cingulate cortex (ACC) and left dorsolateral prefrontal cortex (DLPFC) (Figure 1). Voxel dimensions for both ROIs were 4 cm × 2 cm × 3 cm, resulting in a nominal size of 24 cc. Shimming was performed using the manufacture automated shimming routine (AUTOSHIM), to achieve a full-width at half maximum (FWHM) ≤10 Hz. MRS spectra were obtained by using the interleaved J-difference editing MEGA-PRESS method, as previously described (34, 35). The frequencies of the editing pulses alternated between editing “on” and editing “off” which were centered at 4.56 and 7.5 ppm, respectively. Upon subtraction of the “on” and “off” conditions, the edited-GSH resonance at 2.95 ppm is observed (Figure 1). Data acquisition parameters were: echo time (TE) = 68 ms; recovery time (TR) = 1.5 s; spectral width = 5000 Hz; number of points per spectra = 4096; NEX = 8; total averages acquired = 512; editing RF pulse width = 14.4 ms; scan time = 13:12 (min:s).

Interactive Data Language (IDL)-based software [XsOs-NMR (36)] was used to process the edited-GSH and the unsuppressed water spectra. Raw MRS data from each coil was combined in the time domain based on coil sensitivity (37) from the unsuppressed water signal, weighted by the sum of squares of the signal intensities from each coil. The data was spectrally apodised with a 3 Hz Gaussian filter and then zero filled to 8,192 points, prior to being Fourier transformed. Frequency alignment, additional manual phasing and baseline correction was performed on the data prior to fitting. Edited-GSH and unsuppressed water peaks were modeled using pseudo-voight fitting functions and then fitted in the frequency domain using a highly optimized public-domain Levenberg-Marquardt non-linear least-squares minimization routine, MPFIT (38). Due to the manual phasing and baseline correction that require user input, the data set was randomized and processed two more times by the same user, resulting in three measurements per scan. The measurements were averaged together, and the standard deviation (SD) was calculated. The coefficient of variability (%CV = SD/average) was used to assess the reproducibility of the user. Histograms of the %CV could be used to identify outliers. We found that a %CV threshold of 10% yielded good results and excluded spectra that were visibly of poor quality. The editing-OFF spectra were parsed, frequency corrected, and combined using the FID-A toolkit (39) and was then analyzed using LCModel [version 6.3-0E] (40) using an in-house basis set. SPM12 (41) was used for tissue segmentation of the T1 images. MRS voxel and image registration and fractional tissue within voxel was performed using Gannet and SPM12 (42, 43); data were inspected for correct voxel placement.

### 2.5. Statistical analysis

Descriptive statistics (mean/median, standard deviation interquartile ranges) were calculated for participant demographics and medical history (e.g., age, sex, race, and questionnaire scores). Group differences were evaluated by independent samples *T*-tests, Mann Whitney *U* tests, or Chi square tests where appropriate. Independent samples *T*-tests were employed to evaluate group differences in GSH with follow up tests completed controlling for age and smoking status. Additional step-wise univariate regressions were completed to evaluate possible variance in brain GSH explained by age, smoking status and MA use characteristics/symptom scores. Two-tailed Pearson correlations were employed to evaluate possible correlations between GSH and MA use characteristics, questionnaire scores, and cognitive outcomes. Independent samples *T*-tests were employed to evaluate group differences in peripheral immunoinflammatory blood biomarkers; multiple comparisons were corrected for at an FDR of 0.05. Pearson correlations were employed to evaluate the relationship between blood biomarkers and brain GSH. Since this research question was exploratory, we did not apply a correction for multiple comparisons. Next, interacting variables between standardized blood biomarker data and group status were computed (biomarker * group) and entered into a linear regression model to predict brain GSH. Due to small sample size, β coefficients and 95% confidence intervals are reported. All statistical analysis was conducted using IBM SPSS Statistics 27 (Armonk, NY, USA).

## 3. Results

Twenty-three HC and 14 MA users were enrolled into the study and completed an MRI scan where GSH spectra was acquired in the ACC and DLPFC. After MRI scan quality control, 21 HC and 13 MA users data were analyzed in the ACC, while 19 HC and 10 MA users’ data were analyzed in the DLPFC. Peripheral blood data was collected and analyzed in all 14 MA users and 13 HC participants.

Participant demographics are reported in Table 1. Study groups were well matched except for cigarette use: MA users reported significantly more cigarette use (*p* = 0.004). All MA users tested positive for MA (urine analysis) on MRI scan day and reported abstaining from MA ∼ 1.7 days prior to their MRI scan. The most common route of MA administration was smoking (*n* = 8), and four participants reported multiple routes. MA users reported using MA for ∼ 11 years and current patterns of use were ∼ 5 days or ∼ 2 grams per week. All MA use characteristics are reported in Table 2. Mean blood levels of MA and AM were ∼231 nmol/mL and 45.6 nmol/mL, respectively [measured at PET scan as part of a larger study (10)].

**TABLE 1:** Participant demographics.

Characteristic	Controls *n* = 21		Meth *n* = 14 ACC (*n* = 13); DLPFC (*n* = 10)		*P*-value
Age (range)	32.5 (19–53)		39.6 (22–71)		0.124^A^
Sex, M/F (%female)	11/10 (48)		10/4 (29)		0.211^B^
NIH race, *n* (%Caucasian)	10 (47)		7 (50)		0.240^C^
Years of education	15.5 ± 1.9		13.9 ± 3.17		0.076^A^
Body mass index	24.4 ± 4.31		23.7 ± 3.74		0.659^A^
Alcoholic drinks/week	2.95 ± 3.4		2.5 ± 3.83		0.740^A^
Daily smoker N (% yes)	3 (12)		10 (71)		<0.001^C^
Cigarettes/day	1.6 ± 4.10		13.9 ± 13.26		0.004^A^
Positive urine toxicology (on scan day)	THC	2	THC	2	
	Cocaine	0	Cocaine	2	
	Opiates	0	Opiates	2	
	AMP	0	AMP	14	
	Meth	0	Meth	14	
	MTD	0	MTD	3	
	Morphine	0	Morphine	2	
	BZO	0	BZO	4	
	OXI	0	OXI	0	

A total of 14 participants with methamphetamine (MA) use disorder were scanned for the study. Thirteen had usable data in the anterior cingulate cortex (ACC), 10 had usable data in the left dorsolateral prefrontal cortex (DLPFC). This table presents demographics for all 14 participants.

^A^Independent samples *T*-test.

^B^Chi square test.

^C^Likelihood ratio test.

MDMA, 3,4-methylenedioxymethamphetamine; AM, amphetamine; BZO, benzodiazepine; MTD, methadone; OXI, oxycodone; THC, tetrahydrocannabinol.

**TABLE 2:** Methamphetamine (MA) use characteristics (*n* = 14).

Characteristics	Mean SD
Age of onset (years)	28.9 ± 15.03
Duration of use (years)	10.6 ± 8.7
Frequency (days/week)	5.04 ± 2.3
Amount used/week (grams)	1.84 ± 1.7
**Route of MA administration**	
Smoked	8
IV	2
Nasal	3
Multiple routes of administration	4
Severity of dependence scale (range)	7.38 (5–11)
**Polysubstance (hair positive)**	
MA/AM	14
Cocaine	11
Opiates	3
THC-COOH	6
MDMA	1
MA abstinence before MRI scan (days)	1.77 ± 1.5
**MA withdrawal**	
ASSA (range)	51.5 (24–80)
AWQ (range)	20.6 (8–36)
**DSQ (range)**	
Total	119.4 (75–179)
Reinforcement	19.6 (10–37)
Strong desire	24.6 (10–55)
Mild desire	17.4 (8–28)
Control	9.1 (2–14)

A total of 14 participants with MA use disorder were scanned for the study. Thirteen had usable data in the anterior cingulate cortex (ACC), 10 had usable data in the left dorsolateral prefrontal cortex (DLPFC). This table presents use characteristics for all 14 participants. MDMA, 3,4-methylenedioxymethamphetamine; AM, amphetamine; ASSA, amphetamine selective severity assessment; AWQ, amphetamine withdrawal questionnaire; DSQ, desire for speed questionnaire; THC, tetrahydrocannabinol.

### 3.1. No group differences in brain GSH

**FIGURE 2:**
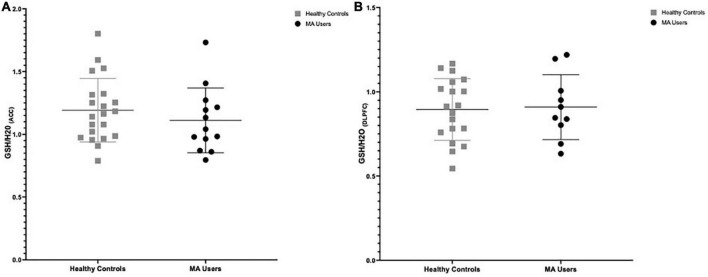
Group differences in glutathione (GSH) between methamphetamine (MA) users (black circles) and HA (gray squares). **(A)** GSH/H20 in the anterior cingulate cortex (ACC): No group differences in GSH between MA users (*n* = 13, mean: 1.111) and HC (*n* = 21, mean:1.192) participants (–7.3% difference, *p* = 0.375). **(B)** GSH/H20 in the dorsolateral prefrontal cortex (DLPFC): No group differences in GSH between MA users (*n* = 10, mean: 0.917) and HC (*n* = 19, mean: 0.895) participants (% difference 1.6%, *p* = 0.847).

There were no significant group differences in GSH in the ACC (%difference −7.3%, *p* = 0.375) nor in the DLPFC (%difference +1.6%, *p* = 0.847) (see Figure 2). Furthermore, smoking status (daily smokers *n* = 10 MA/3 HC; *p* < 0.001) and age were not significant covariates in this model (*p* > 0.2) and there were no significant differences in brain GSH between sexes in the ACC (*p* = 0.8) or DLPFC (*p* = 0.5). Co-use of cannabis (*p* > 0.8) or cocaine (*p* > 0.3), as detected in hair toxicology, did not influence GSH concentrations in the ACC or DLPFC. Stepwise univariate regressions did not identify any significant factors or MA use characteristics that accounted for the variance in brain GSH in the ACC nor DLPFC. Finally, there were no relationships between GSH and MA use characteristics, questionnaire scores, or cognitive outcomes (*p* > 0.2).

### 3.2. Relationship between peripheral blood biomarkers and brain GSH

**FIGURE 3:**
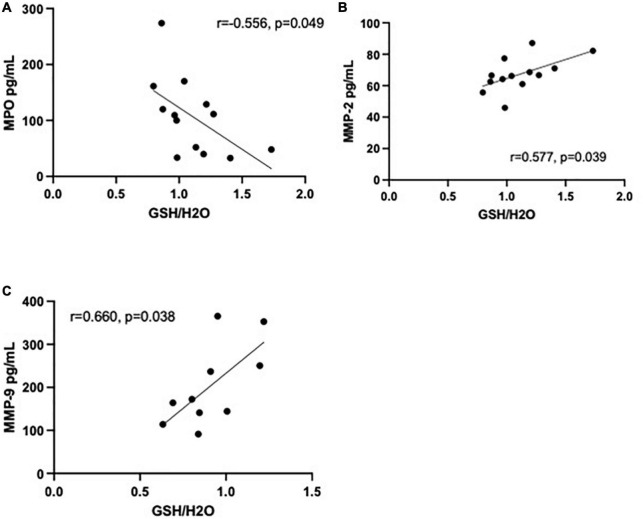
Correlations between immunoinflammatory biomarkers and glutathione (GSH) in methamphetamine users (black circles). **(A)** Myeloperoxidase (MPO) (pg/mL) is negatively correlated with GSH (*r* = −0.556, *p* = 0.049, *n* = 13) in the anterior cingulate cortex (ACC) and **(B)** matrix metalloproteinase (MMP)-2 (pg/mL) is positively correlated with GSH (*r* = 0.577, *p* = 0.039, *n* = 13) in the ACC. **(C)** MMP-9 (pg/mL) was positively correlated with GSH (*r* = 0.660, *p* = 0.038, *n* = 10) in the dorsolateral prefrontal cortex (DLPFC).

Data of 25 immunoinflammatory blood biomarkers were available for 13 HC and 14 MA users in our study (see Supplementary Table 2). Concentrations of 19 biomarkers (out of 25) were significantly elevated in MA users compared to HC. Increases in 17 biomarkers remained significant after correcting for multiple comparisons (FDR 0.05). Of the 25 biomarkers measured, statistically significant correlations with GSH in the MA users were limited to matrix metalloproteinase (MMP)-2 (*r* = 0.577, *p* = 0.039) and myeloperoxidase (MPO) (*r* = −0.556, *p* = 0.049) in the ACC and MMP-9 in the DLPFC (*r* = 0.660, *p* = 0.038) (see Figure 3). Note, if an FDR of 0.05 were applied, correlations would not have survived correcting for multiple comparisons. All correlations between brain GSH and peripheral immunoinflammatory markers can be found in Supplementary Tables 3, 4. Additionally, MPO [β = −0.024 CI (−0.046 to −0.002)] and MMP-2 [β = 0.025 CI (−0.001 to 0.05)] demonstrated the strongest interaction effect between study groups in predicting GSH in the ACC. MMP-9 [β = −0.013 CI (−0.002 to 0.028)] demonstrated a moderate interaction effect between study groups in predicting GSH in the DLPFC. All interaction coefficients can be found in Supplementary Figures 1, 2.

## 4. Discussion

We did not observe any statistically significant differences in GSH concentrations between HC and MA users in the ACC and DLPFC, or any relationships between brain GSH levels and drug use characteristics or behavioral outcomes of the participants.

Our findings differ from those of Su et al. (28) who reported markedly (35%) increased GSH (in left DLPFC) in patients with MA-related SUD compared to HC. However, the different metabolite acquisition methods should be considered when interpreting findings: we employed J-editing MEGA-PRESS to quantify brain GSH, while Su et al., (28) employed PRESS. As discussed above, research has demonstrated that MEGA-PRESS acquisition is capable of quantifying GSH at physiological concentrations while PRESS acquisition cannot (29). Additionally, differences in study design and participants could have contributed to the opposing findings. First, time since last MA use might have affected GSH concentrations. In this regard, the MA users in our study abstained from MA for ∼1.7 days prior to their MRI scan, and all tested positive for MA on urine toxicology at the day of their scan, but MA abstinence time was not reported in the Su et al. (28) investigation. Use of other drugs could also have affected the GSH outcome measure. Su et al. (28) do not report information on co-use of other drugs, whereas in our study 11 and 6 both tested positive for cocaine and THC on hair toxicology, respectively. In an earlier study, however, we reported normal GSH levels in autopsied brain of chronic cocaine users (44).

### 4.1. Negative GSH finding

We previously reported evidence of oxidatively damaged lipids in autopsied striatum and frontal cortex of chronic MA users (26), suggesting the possibility that brain levels of the antioxidant GSH might be overutilized during oxidative stress. However, our negative neuroimaging GSH finding in the living brain is generally consistent with our previous observation in autopsied brains of MA users in which statistically significant GSH changes were not observed (27). The simplest explanation for the null GSH finding in the present study is that oxidative stress processes following chronic MA use in the current study’s participants might not have been sufficient to substantially alter brain GSH levels. This could be because the doses at which our participants were using (∼2 grams/week) were insufficient to cause overutilization and subsequent depletion of GSH. It may also be relevant that in our preclinical study, in which rats were administered neurotoxic levels of MA (4 × 20 mg/kg every 5 h) in binge cycle pattern, only a modest 17% reduction in striatal GSH was observed (18).

### 4.2. Immunoinflammatory blood biomarker findings

Our finding of increased concentrations of immunoinflammatory blood biomarkers in MA users is in line with previous research (13), and suggests chronic MA use involves systemic inflammatory processes. There is also research implicating central inflammation in MA use disorder (45). Considering the blood brain barrier (BBB) might be disrupted in MA use (46) (increased VEGF in the current study’s MA group may suggest this), it is unclear if peripheral and central inflammation occur simultaneously or if one propagates the other. The origin (peripheral circulation vs. brain) of these blood biomarkers is also unknown and we cannot know if the detected increase in immunoinflammatory blood biomarker concentrations is related to systemic inflammation or entered into the periphery from the brain *via* a leaky BBB. Our preliminary finding that concentrations of peripheral immunoinflammatory blood biomarkers, specifically MMP-2, MPO, and MMP-9, correlate with brain GSH tentatively suggest that systemic inflammation might be related to MA induced oxidative stress processes. This initial finding is supported by the observed group by biomarker interaction effect on brain GSH levels; specifically, the relationship between MPO, MMP2, MMP-9, and MCP-1 and brain GSH appears to be specific to the MA use group. Research has implicated MMPs and MCP-1 in methamphetamine use (47, 48). In their preclinical work, Mizoguchi et al. (47) suggest increased expression of MMP-2 and MMP-9 may be related to synaptic plasticity and functional alterations following chronic MA exposure. Additionally, the authors (47) observed a relationship between synaptic dopamine and MMP-2/9, suggesting a role for these proteins in dopamine transport during MA use. Loftis et al. (48) observed a relationship between plasma MCP-1 concentrations and language fluency in participants recovering from MA dependance and Sevigny et al. (49) reported a relationship between cerebrospinal fluid (CSF) MCP-1 and onset of dementia in humans living with human immunodeficiency virus (HIV). MPO plays a role in the formation of reactive oxidant species and has been implicated in neurodegeneration (50), a common outcome following chronic MA use. This is the first study (to our knowledge) to report on relationships between peripheral blood biomarkers and brain metabolites in methamphetamine users and further research is needed to understand the role the role they have in MA use and related oxidative stress processes.

### 4.3. Limitations

Limitations of our study include a small sample size and limited number of brain regions examined. A larger sample size would have enabled better controlling for confounders such as age, smoking status, and polysubstance use (e.g., cocaine and THC) without sacrificing statistical power. The large number of polysubstance users (e.g., *n* = 11/14 cocaine, *n* = 6/14 THC) enrolled in the current MA cohort could have influenced findings and make it difficult to conclude findings are methamphetamine related. While the ACC and DLPFC regions were investigated in this study, it would also be interesting to assess other brain regions related to addiction including the striatum and insula.

### 4.4. Conclusion and possible next steps

Our overall findings to date tentatively suggest that although oxidative damage might occur in the brain of some MA users, this may not be severe enough to be accompanied by depletion of the antioxidant GSH. Our results further support growing evidence for the dysregulation of multiple immunoinflammatory mediators/pathways in chronic MA use. Future research might evaluate further the possible relationship between brain levels of GSH and blood immune markers affected in MA users.

## Data availability statement

The raw data supporting the conclusions of this article will be made available by the authors, without undue reservation.

## Ethics statement

The studies involving human participants were reviewed and approved by Centre for Addiction and Mental Health (CAMH) Research Ethics Board. The patients/participants provided their written informed consent to participate in this study.

## Author contributions

IB, SK, JT, SR, and SC contributed to the conception of design and data interpretation. SW, SJ, TM, JW, SH, JT, PT, and SC contributed to the data acquisition and data analysis, and interpretation. SW, SJ, IB, and SK drafted the manuscript. All authors critically revised the manuscript and provided full approval of version to be published.
