# Supplementary material for: Imaging oxidative stress in brains of chronic methamphetamine users: A combined 1H-magnetic resonance spectroscopy and peripheral blood biomarker study
Source: Front Psychiatry. 2023 Jan 10;13:1070456. doi: 10.3389/fpsyt.2022.1070456 (PMC9871559; doi:10.3389/fpsyt.2022.1070456)
Supplement: Supplementary file 1 [file Data_Sheet_1.docx]

Supplementary Material

# Supplementary Data

None.

# Supplementary Figures and Tables

| **Table S1: Questionnaires and Cognitive Tasks Administered to Participants** | | |
| --- | --- | --- |
| *Questionnaires* | Description | Reference |
| Amphetamine Withdrawal Questionnaire | Assesses methamphetamine withdrawal. Possible range of scores is 0–40 with higher score indicating greater severity. | (1) |
| Amphetamine Selective Severity Assessment Scale | Assesses methamphetamine withdrawal. Each item is scored on a 0 to 7 scale. For paired items 1 (hyperphagia) and 2 (hypophagia) and paired items 11 (hyposomnia) and 12 (hypersomnia), if one item is > 0, the other must equal 0. The total score is a sum of items 1–22, with a range of 0–140. Higher scores indicate greater intensity or frequency of withdrawal symptoms. | (2) |
| Desire for Speed Questionnaire | Assesses methamphetamine craving. 40 item scale with likert-type scale where participants score each item 1 to 7 as to how strongly they agreed or disagreed with each statement | (3) |
| Severity of Dependence Scale | Assesses methamphetamine dependance. A 5 item likert type sale where participants score 0-4 how strongly they agree with each statement. | (4) |
| *Cognitive Task* | Cognition Assessed | Reference |
| Hopkins Verbal Learning Test | Verbal Memory | (5) |
| Digit Span Test span – Wechsler Memory Scale, 3rd Ed | Working Memory | (6) |
| Trail Making A-B | Speed of processing | (7) |
| The Purdue Pegboard Test | Motor Speed and Control | (13) |
| Connor’s Continuous Performance Test - II | Attention | (8) |
| Kirby Delayed Discounting | Response inhibition/decision making | (9) |
| Spatial Delayed Response Task | Visuo-spatial memory | (10) |
| Stop Signal Task | Response inhibition/decision making | (11) |

| **Table S2: Group differences in immunoinflammatory blood biomarker concentrations** | | | |
| --- | --- | --- | --- |
| **Protein** | **Group** | **Concentration** | **P Value** |
| Eotaxin | Healthy Controls | 128.18±55.5 | <0.001 |
|  | MA users | 445.58±232.3 |  |
| Eotaxin-3 | Healthy Controls | 11.07±4.9 | <0.001 |
|  | MA users | 27.39±9.1 |  |
| IP-10 | Healthy Controls | 216.11±162.3 | <0.001 |
|  | MA users | 736.44±378.6 |  |
| MCP-1 | Healthy Controls | 213.91±109.7 | <0.001 |
|  | MA users | 469.48±95.9 |  |
| MCP-4 | Healthy Controls | 107.36±27.2 | <0.001 |
|  | MA users | 226.27±32.1 |  |
| MDC | Healthy Controls | 685.24±171.9 | <0.001 |
|  | MA users | 1620.89±430.5 |  |
| MIP- α | Healthy Controls | 31.21±17.1 | <0.001 |
|  | MA users | 115.76±38.2 |  |
| MIP-1β | Healthy Controls | 74.71±27.8 | 0.001 |
|  | MA users | 116.41±31.1 |  |
| TARC | Healthy Controls | 140.16±64.3 | 0.002 |
|  | MA users | 384.89±240.3 |  |
| IFN-γ | Healthy Controls | 2.63±1.8 | 0.005 |
|  | MA users | 10.18±8.2 |  |
| IL-10 | Healthy Controls | .28±0.22 | 0.051 |
|  | MA users | .67±0.65 |  |
| IL-6 | Healthy Controls | .39±0.28 | <0.001 |
|  | MA users | 2.72±1.4 |  |
| IL-8 | Healthy Controls | 4.38±1.6 | <0.001 |
|  | MA users | 9.03±2.9 |  |
| TNF-α | Healthy Controls | 2.59±1.3 | 0.727 |
|  | MA users | 2.77±1.3 |  |
| IL-7 | Healthy Controls | 9.23±6.6 | 0.006 |
|  | MA users | 16.28±5.3 |  |
| TNF- β | Healthy Controls | 0.49±0.261 | 0.525 |
|  | MA users | 0.41±0.265 |  |
| VEGF | Healthy Controls | 51.42±27.2 | <0.001 |
|  | MA users | 211.96±98.5 |  |
| CRP | Healthy Controls | 1.28±0.57 | 0.096 |
|  | MA users | 2.06±1.6 |  |
| MMP-1 | Healthy Controls | 16.85±5.4 | 0.008 |
|  | MA users | 29.02±14.2 |  |
| MMP-2 | Healthy Controls | 65.63±11.6 | 0.760 |
|  | MA users | 66.94±10.4 |  |
| MMP-3 | Healthy Controls | 13.45±7.0 | 0.184 |
|  | MA users | 16.98±6.3 |  |
| MMP-9 | Healthy Controls | 97.04±31.2 | 0.002 |
|  | MA users | 185.61±85.9 |  |
| MMP-10 | Healthy Controls | 1.25±0.51 | 0.011 |
|  | MA users | 2.00±0.84 |  |
| BDNF | Healthy Controls | 12.14±3.4 | 0.004 |
|  | MA users | 18.5±6.4 |  |
| MPO | Healthy Controls | 57.05±31.0 | 0.037 |
|  | MA users | 102.24±68.1 |  |
| MA users n=14; HC n=13  Acronyms: Brain derived neurotrophic factor BDNF); c-reactive protein (CRP); Interferon gamma (IFN-γ ); Interferon gamma-induced protein (IP)-10; Interleukin (IL); Macrophage-derived chemokine (MDC); Macrophage Inflammatory Protein(MIP); Matrix Metallopeptidase. (MMP); Monocyte chemoattractant protein (MCP); Myeloperoxidase (MPO) thymus- and activation-regulated chemokine (TARC); Tumor necrosis factor (TNF); Vascular endothelial growth factor (VEGF). | | | |

| **Table S3: Correlations between peripheral blood biomarkers and GSH in ACC** | | | |
| --- | --- | --- | --- |
| ***ACC*** | | | |
| **Marker** | **Group** | **R** | **P value** |
| Eotaxin | MA | 0.009 | 0.978 |
|  | HC | 0.487 | 0.091 |
| Eotaxin-3 | MA | 0.081 | 0.794 |
|  | HC | -0.362 | 0.304 |
| IP-10 | MA | -0.108 | 0.724 |
|  | HC | 0.274 | 0.365 |
| MCP-1 | MA | -0.034 | 0.913 |
|  | HC | 0.398 | 0.177 |
| MCP-4 | MA | 0.377 | 0.205 |
|  | HC | 0.097 | 0.763 |
| MDC | MA | -0.330 | 0.271 |
|  | HC | 0.071 | 0.816 |
| MIP-α | MA | 0.034 | 0.911 |
|  | HC | -0.761 | 0.028* |
| MIP-β | MA | 0.196 | 0.521 |
|  | HC | 0.569 | 0.042* |
| TARC | MA | 0.456 | 0.117 |
|  | HC | 0.425 | 0.147 |
| IFN-λ | MA | -0.352 | 0.238 |
|  | HC | 0.382 | 0.246 |
| IL-10 | MA | -0.27 | 0.929 |
|  | HC | 0.620 | 0.032* |
| IL-6 | MA | -0.249 | 0.412 |
|  | HC | 0.520 | 0.083 |
| IL-8 | MA | -0.483 | 0.094 |
|  | HC | 0.112 | 0.714 |
| TNF-α | MA | -0.275 | 0.362 |
|  | HC | -0.19 | 0.952 |
| IL-7 | MA | 0.425 | 0.148 |
|  | HC | 0.391 | 0.209 |
| TNF-β | MA | -0.129 | 0.673 |
|  | HC | 0.619 | 0.138 |
| VEGF | MA | 0.056 | 0.857 |
|  | HC | 0.226 | 0.458 |
| CRP | MA | -0.190 | 0.534 |
|  | HC | -0.332 | 0.268 |
| MMP-1 | MA | -.319 | 0.288 |
|  | HC | 0.203 | 0.505 |
| MMP-2 | MA | 0.577 | 0.039* |
|  | HC | 0.306 | 0.310 |
| MMP-3 | MA | -0.242 | 0.425 |
|  | HC | -0.109 | 0.722 |
| MMP-9 | MA | 0.527 | 0.064 |
|  | HC | -0.30 | 0.922 |
| MMP-10 | MA | 0.057 | 0.854 |
|  | HC | -0.509 | 0.076 |
| BDNF | MA | 0.078 | 0.800 |
|  | HC | -0.306 | 0.309 |
| MPO | MA | -0.556 | 0.049* |
|  | HC | 0.134 | 0.661 |
| MA users n=13; HC n=13  Acronyms: Brain derived neurotrophic factor BDNF); c-reactive protein (CRP); Interferon gamma (IFN-γ ); Interferon gamma-induced protein (IP)-10; Interleukin (IL); Macrophage-derived chemokine (MDC); Macrophage Inflammatory Protein(MIP); Matrix Metallopeptidase. (MMP); Monocyte chemoattractant protein (MCP); Myeloperoxidase (MPO) thymus- and activation-regulated chemokine (TARC); Tumor necrosis factor (TNF); Vascular endothelial growth factor (VEGF). | | | |

| **Table S4: Correlations between peripheral blood biomarkers and GSH in DLPFC** | | | |
| --- | --- | --- | --- |
| ***DLPFC*** | | | |
| **Marker** |  | **R** | **P** |
| Eotaxin | MA | -0.182 | 0.615 |
|  | HC | -0.526 | 0.096 |
| Eotaxin-3 | MA | 0.316 | 0.374 |
|  | HC | 0.033 | 0.932 |
| IP-10 | MA | -0.118 | 0.746 |
|  | HC | -0.430 | 0.187 |
| MCP-1 | MA | -0.126 | 0.728 |
|  | HC | -0.734 | 0.010* |
| MCP-4 | MA | -0341 | 0.335 |
|  | HC | -0.072 | 0.843 |
| MDC | MA | 0.516 | 0.127 |
|  | HC | 0.355 | 0.284 |
| MIP-α | MA | -0.108 | 0.767 |
|  | HC | -0.284 | 0.533 |
| MIP-β | MA | 0.125 | 0.731 |
|  | HC | -0.286 | 0.486 |
| TARC | MA | 0.047 | 0.898 |
|  | HC | 0.021 | 0.952 |
| IFN-γ | MA | 0.177 | 0.625 |
|  | HC | -0.194 | 0.617 |
| IL-10 | MA | 0.192 | 0.596 |
|  | HC | -0.119 | 0.743 |
| IL-6 | MA | -0.067 | 0.854 |
|  | HC | -0.637 | 0.048* |
| IL-8 | MA | -0.214 | 0.552 |
|  | HC | -0.021 | 0.952 |
| TNF-α | MA | 0.358 | 0.309 |
|  | HC | -0.295 | 0.378 |
| IL-7 | MA | 0.271 | 0.450 |
|  | HC | -0.303 | 0.366 |
| TNF-β | MA | -0.063 | 0.863 |
|  | HC | -0.520 | 0.232 |
| VEGF | MA | 0.104 | 0.776 |
|  | HC | -0.163 | 0.631 |
| CRP | MA | 0.512 | 0.131 |
|  | HC | 0.244 | 0.470 |
| MMP-1 | MA | 0.135 | 0.710 |
|  | HC | -0.529 | 0.094 |
| MMP-2 | MA | -0.289 | 0.419 |
|  | HC | 0.00 | 0.999 |
| MMP-3 | MA | -0.022 | 0.952 |
|  | HC | -0.095 | 0.781 |
| MMP-9 | MA | 0.660 | 0.038* |
|  | HC | 0.016 | 0.963 |
| MMP-10 | MA | -0.376 | 0.285 |
|  | HC | -0.120 | 0.726 |
| BDNF | MA | 0.110 | 0.761 |
|  | HC | 0.267 | 0.428 |
| MPO | MA | 0.006 | 0.988 |
|  | HC | 0.141 | 0.680 |
| MA users n=10; HC n=13  Acronyms: Brain derived neurotrophic factor BDNF); c-reactive protein (CRP); Interferon gamma (IFN-γ ); Interferon gamma-induced protein (IP)-10; Interleukin (IL); Macrophage-derived chemokine (MDC); Macrophage Inflammatory Protein(MIP); Matrix Metallopeptidase. (MMP); Monocyte chemoattractant protein (MCP); Myeloperoxidase (MPO) thymus- and activation-regulated chemokine (TARC); Tumor necrosis factor (TNF); Vascular endothelial growth factor (VEGF). | | | |

## Supplementary Figures

**Supplementary Figure 1**: Group*Biomarker Interaction Coefficients (95 CI) in the ACC: β coefficients (95 CI) of Group*Biomarker interaction in predicting GSH concentrations in the ACC.
Acronyms: Brain derived neurotrophic factor BDNF); c-reactive protein (CRP); Interferon gamma (IFN-γ ); Interferon gamma-induced protein (IP)-10; Interleukin (IL); Macrophage-derived chemokine (MDC); Macrophage Inflammatory Protein(MIP); Matrix Metallopeptidase. (MMP); Monocyte chemoattractant protein (MCP); Myeloperoxidase (MPO) thymus- and activation-regulated chemokine (TARC); Tumor necrosis factor (TNF); Vascular endothelial growth factor (VEGF).

**Supplementary Figure 2**: Forest plot listing the measure of blood biomarkers sampled. The confidence intervals illustrate the Group*Biomarker Interaction (95 CI) in the DLPFC: β coefficients (95 CI) of Group*Biomarker interaction in predicting GSH concentrations in the ACC.
Acronyms: Brain derived neurotrophic factor BDNF); c-reactive protein (CRP); Interferon gamma (IFN-γ ); Interferon gamma-induced protein (IP)-10; Interleukin (IL); Macrophage-derived chemokine (MDC); Macrophage Inflammatory Protein(MIP); Matrix Metallopeptidase. (MMP); Monocyte chemoattractant protein (MCP); Myeloperoxidase (MPO) thymus- and activation-regulated chemokine (TARC); Tumor necrosis factor (TNF); Vascular endothelial growth factor (VEGF).

**3 References**

1. Srisurapanont M, Jarusuraisin N, Jittiwutikan J. Amphetamine withdrawal: I. Reliability, validity and factor structure of a measure. Australian & New Zealand Journal of Psychiatry. 1999;33(1):89-93.

2. Kampman KM, Volpicelli JR, McGinnis DE, Alterman AI, Weinrieb RM, D'Angelo L, et al. Reliability and validity of the Cocaine Selective Severity Assessment. Addict Behav. 1998;23(4):449-61.

3. James D, Davies G, Willner P. The development and initial validation of a questionnaire to measure craving for amphetamine. Addiction. 2004;99(9):1181-8.

4. Gossop M, Darke S, Griffiths P, Hando J, Powis B, Hall W, et al. The Severity of Dependence Scale (SDS): psychometric properties of the SDS in English and Australian samples of heroin, cocaine and amphetamine users. Addiction. 1995;90(5):607-14.

5. Shapiro AM, Benedict RH, Schretlen D, Brandt J. Construct and concurrent validity of the Hopkins Verbal Learning Test-revised. Clin Neuropsychol. 1999;13(3):348-58.

6. Elwood RW. The Wechsler Memory Scale-Revised: psychometric characteristics and clinical application. Neuropsychol Rev. 1991;2(2):179-201.

7. Reitan RM. Validity of the Trail Making Test as an indicator of organic brain damage. Perceptual and motor skills. 1958;8(3):271-6.

8. Conners C, Staff M. Continuous Performance Test II (CPT-II) computer programs for Windows technical guide and software manual. Noth Tonawada, New York: Multi-Health Systems. 2000.

9. Kirby KN, Petry NM, Bickel WK. Heroin addicts have higher discount rates for delayed rewards than non-drug-using controls. J Exp Psychol Gen. 1999;128(1):78-87.

10. Hershey T, Perantie DC, Warren SL, Zimmerman EC, Sadler M, White NH. Frequency and timing of severe hypoglycemia affects spatial memory in children with type 1 diabetes. Diabetes Care. 2005;28(10):2372-7.

11. Nichols SL, Waschbusch DA. A review of the validity of laboratory cognitive tasks used to assess symptoms of ADHD. Child Psychiatry Hum Dev. 2004;34(4):297-315.

**
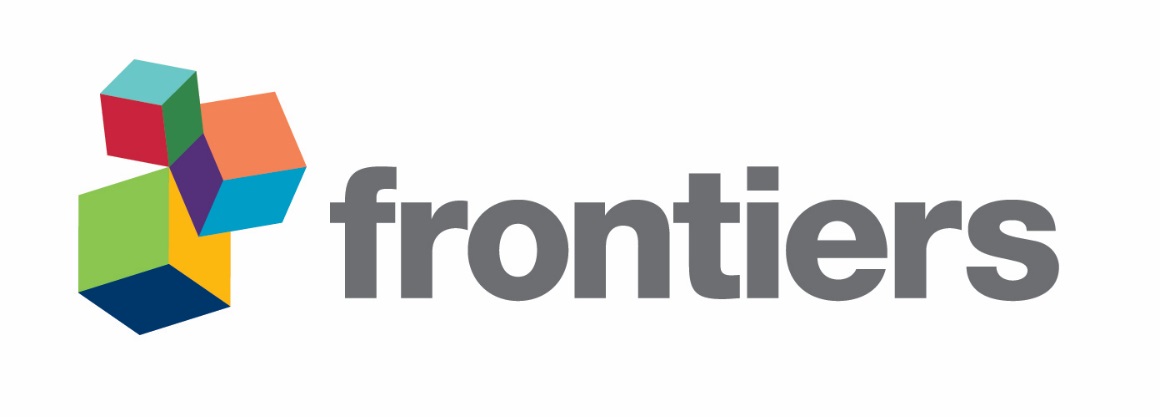
**
